# A tailored maturity model for REDCap-based patient-facing technology: revealing development stages and guiding future evolution

**DOI:** 10.1093/jamiaopen/ooag176

**Published:** 2026-09-10

**Authors:** Zhengcan Xie, Yuheng Shi, Eric Yang, Yun Jiang, Yang Gong

**Affiliations:** D. Bradley McWilliams School of Biomedical Informatics, The University of Texas Health Science Center at Houston, Houston, TX 77030, United States; D. Bradley McWilliams School of Biomedical Informatics, The University of Texas Health Science Center at Houston, Houston, TX 77030, United States; D. Bradley McWilliams School of Biomedical Informatics, The University of Texas Health Science Center at Houston, Houston, TX 77030, United States; School of Nursing, University of Michigan, Ann Arbor, MI 48109, United States; D. Bradley McWilliams School of Biomedical Informatics, The University of Texas Health Science Center at Houston, Houston, TX 77030, United States

**Keywords:** REDCap, patient-centered care, patient-facing technology (PFT), electronic patient-reported outcomes (ePRO)

## Abstract

**Objectives:**

This integrative review aims to analyze the REDCap-based patient-facing technologies (PFTs) and propose a tailored maturity model to characterize their development stages, challenges, and future evolution toward intelligent, proactive care.

**Materials and Methods:**

Following the Preferred Reporting Items for Systematic Reviews and Meta-Analyses (PRISMA) guidelines, a focused search of PubMed, Web of Science, and Embase identified relevant studies. Data were analyzed using aggregate and thematic synthesis. Combining literature evidence with pilot case insights, a tailored maturity model was developed to define the distinct stages of REDCap-based PFT development.

**Results:**

The proposed model progresses from Data Collection (Tier 1) and System Integration (Tier 2) to Personalized Insights (Tier 3), culminating in a conceptual Intelligence Hub (Tier 4). Analysis of 14 studies (2016-2026) reveals a rapidly evolving field, with a surge in publications since 2021 (*n* = 12, 86%). Most included studies were US-based (*n* = 11, 79%), primarily pilot tests (*n* = 6) and system design studies (*n* = 5). Most implementations (*n* = 10, 71%) remain at early maturity stages (Tiers 1 and 2), with only a minority (*n* = 4, 29%) achieving Personalized Insights (Tier 3).

**Conclusion:**

These findings underscore the promise of REDCap-based PFTs but reveal a critical gap between current capabilities and the advanced Intelligence Hub envisioned in the maturity model. Applying the tailored maturity model highlights priorities for advancing research, including rigorous, longitudinal evaluations and strategies to achieve higher levels of integration and optimization.

## Introduction

Engaging patients in managing their chronic conditions is increasingly central to modern healthcare, driving a shift from disease-focused management to patient-centered care.^1^ This transition emphasizes individual preferences and needs and leverages digital technologies to provide near real-time health insights, enhancing safety and quality of life through timely reporting and response.^2^

Patient-facing technologies (PFTs) are digital tools that can interact directly with patients and collect their real-time health data—often in the form of electronic patient-reported outcomes (ePROs)—to improve engagement and healthcare outcomes. PFTs range from common tools like consumer smartwatches and patient portals that incorporate health tracking into everyday routines,^3^^,^^4^ to prescription digital therapeutics and generative AI health copilots that provide automated, personalized clinical interventions directly to individuals.^5^^,^^6^ These technologies enable patients to report, monitor, and manage health information outside traditional clinical settings.^7^^,^^8^ By facilitating real-time data capture, delivering personalized feedback, and integrating with clinical systems, PFTs empower patient self-management and enhance communication with healthcare providers.

Custom-built PFTs, particularly mobile applications, often face significant challenges that limit their long-term viability and interoperability.^7^ These customized solutions typically require extensive technical expertise for development and ongoing maintenance, leading to high resource demands and increased costs. Frequent updates are necessary to keep pace with evolving operating systems and security standards, which can strain institutional budgets and personnel resources. Furthermore, customized PFTs often lack interoperability with other clinical systems, resulting in fragmented workflows and data silos. These limitations hinder scalability, sustainability, and interoperability, necessitating standardized, platform-based PFT solutions.

Increasingly, institutions have adopted Research Electronic Data Capture (REDCap) as a sustainable alternative for developing PFTs. REDCap and its mobile solutions, REDCap Mobile App and MyCap, support both online and offline data capture for research and clinical operations, offering customizable forms, HIPAA-compliant workflows, and built-in tools for survey distribution and data management.^9^^,^^10^ Unlike custom-built standalone applications, REDCap reduces development complexity through a standardized, scalable framework that integrates with external systems via Application Programming Interfaces (APIs), ensuring interoperability and long-term maintainability.^11^^,^^12^ Its widespread adoption across thousands of institutions worldwide underscores its reliability as a foundation for PFT solutions,^13^ making it an appealing choice for research teams seeking a secure, streamlined approach to data collection and management.

Despite these advantages, REDCap has notable limitations concerning PFTs. Its native features—such as web-based surveys and mobile solutions—offer ‘out-of-the-box’ capabilities for basic data capture, but are limited in advanced data integration and visualization. To build more complex PFTs, REDCap is often used as a secure database, with extensive custom development via its API to interface with other health information systems, such as electronic health records (EHRs), and to support real-time analytics.^14^ This shift from native tools to API-driven integrations introduces technical complexities, emphasizing the need for theoretical frameworks and supplementary tools to progress PFTs from simple data collection to comprehensive, insight-driven solutions.^15^

Maturity models are a theoretical framework initially used in software engineering, providing a phased roadmap for system development that guides progress from disorder to optimization and serves as a standard for evaluation.^16^ As digitization increasingly spreads across the healthcare field, maturity models are widely used to develop health information systems, such as EHRs and telemedicine.^17–19^ Maturity models enable research and clinical teams to benchmark their current position, identify gaps, and plan strategically for progression toward advanced, insight-driven systems. However, existing healthcare maturity models rarely incorporate patient-centered care or rigorous data analysis as core dimensions.^20^ Given PFTs’ focus on helping patients and using multidimensional data, a customized maturity model can provide a clear way to address technical, organizational, and usability issues, making it easier to move from reactive data collection toward proactive, intelligent care.

Accordingly, we propose a tailored maturity model based on a review of existing REDCap-based PFTs. The main functions of this maturity model include: (1) *Classification and positioning:* organizing current PFTs according to the complexity of implementation to provide researchers with a clear overview, (2) *Synthesis of experiences and challenges:* summarizing practical experiences and difficulties and analyzing possible connections, and (3) *Guiding progression paths:* providing a roadmap for researchers on how to evolve from simple data collection tools to advanced intelligent integrated systems. Ultimately, this model serves as a practical guide for future research, facilitating the development of more stable, robust, and sustainable REDCap-based PFTs.

## Methods

This study was conducted and reported in accordance with established guidelines for integrative reviews^21^ and the Preferred Reporting Items for Systematic Reviews and Meta-Analyses (PRISMA) statement.^22^ To ensure the review’s reliability and consistency, 2 authors (Z.X. and Y.G.) conducted the study selection. The primary author (Z.X.) performed data extraction and analysis, which was then reviewed and verified by the senior author (Y.G.). Group discussions involving other coauthors helped clarify ambiguities and reach consensus on final decisions.

### Search strategy

We searched PubMed, Web of Science, and Embase for peer-reviewed articles using keywords related to REDCap, MyCap, and patient-centered systems. No date or language filters were applied (full search strategies are shown in Appendix A).

### Screening and selection criteria

To be included in the final analysis, publications had to: (1) be original, peer-reviewed research detailing system design, development, or implementation and (2) describe a REDCap-based patient-centered system designed for interactive or long-term engagement, with sufficient technical detail. We excluded non-primary literature (eg, reviews, editorials) and studies focusing solely on clinical outcomes or usability without technical details. After deduplication (Appendix B), studies underwent standard title/abstract screening, followed by full-text review to confirm eligibility.

### Data extraction

A structured data extraction process was developed to collect key information from each included study. The data collected were then divided into 2 sections to support further analysis.

1. *Descriptive characteristics:* To summarize the general characteristics of the included studies, the following data points were extracted: (1) author and year, (2) study type, (3) country, (4) setting, (5) domain, (6) sample size, and (7) patient age range.
2. *Qualitative data:* Detailed qualitative notes were recorded for each study across 4 key dimensions: (1) stated objectives, (2) technical approaches, including the classification of whether the system relied on native REDCap functionalities or employed custom API integrations, (3) study outcomes, and (4) implementation barriers.

### Data analysis

The primary objective of the data analysis was to construct a tailored maturity model for PFTs by utilizing the dimensions extracted from the included studies. This process involved 3 main analytical steps: aggregate analysis, thematic synthesis, and model conceptualization.

#### Aggregate analysis

The general characteristics of the included studies were summarized by calculating frequencies and percentages. From these descriptive data, the interplay between study type and geographic region was highlighted to further characterize the maturity and scalability of REDCap-based PFTs.

#### Thematic synthesis

A thematic analysis was conducted to identify commonalities and trends among these studies, laying out the empirical foundation for the maturity model. This involved a 3-stage inductive process:

1. *Open coding.* Each study was read in-depth to identify and code all text segments related to implementation strategies, technical features, objectives, and challenges. The initial coding generated a preliminary list of raw concepts from the literature.
2. *Developing descriptive themes.* The initial codes were iteratively compared, contrasted, and grouped into broader descriptive themes. For example, codes such as “API to EHR” and “Twilio for SMS” were grouped under the descriptive theme of “Integration with External Systems.
3. *Generating the analytical framework.* These studies were synthesized to elucidate the deeper relationships among them.

#### Model conceptualization

A conceptual extrapolation was performed to finalize the model as a guiding roadmap for the clinical and translational research community, along with recommendations for PFT developers and the REDCap consortium. This final step involved comparing the study outcomes with the implementation barriers within the advanced tier, thereby identifying the functional discontinuity between passive reporting systems and emerging automated support systems. The extrapolation was further informed by 2 factors: established values for maximizing patient empowerment and the reflection of our team’s collaboration in the design, development, and evaluation of PFTs.

## Results

Details of the literature search and screening process are shown in the PRISMA flow diagram (Appendix C). The initial database search found 1037 records. After removing 240 duplicates, 797 remained for title and abstract screening. Of these, 740 were excluded for failing to meet the criteria, leaving 57 for the full text review. After reviewing full texts, 46 were excluded. Upon examining reference lists, 5 additional relevant articles were identified. In total, 16 publications representing 14 unique studies were included in the final analysis.

### Characteristics of included studies

To characterize the progression, the foundation of the tailored maturity model was outlined to illustrate the evolution of these REDCap-based PFTs from standalone data collectors (Tier 1) to integrated systems that automate workflows (Tier 2) and advanced platforms that provide personalized insights (Tier 3). The results of the aggregate analysis of the 14 studies are summarized in Table 1. The identified literature spanned 2016-2026, with a notable surge in research activity published since 2021 (*n* = 12; 85.7%). Beyond this temporal trend, other key characteristics include: (1) a clear focus on chronic diseases, which represent the application domain (*n* = 13; 93%), (2) a wide age distribution, covering all age groups, and (3) a majority of these studies focused on 2 fundamental maturity levels (71%): Tier 1 (*n* = 4) and Tier 2 (*n* = 6). A few studies (*n* = 4, 29%) have achieved Tier 3, indicating that these PFTs are evolving toward more sophisticated capabilities.

**Table 1.** General characteristics of included studies grouped by maturity tier.

	Author, year	Study type	Country	Setting	Domain	Sample size	Age (years)
Data collection	Kandula et al. (2016)^23^	Pilot test	USA	Community cohort	Cardiology: atherosclerosis	*N* = 906	40-84
	Huynh et al. (2021)^24^	Impact evaluation	USA	Academic medical center	Urology: prostate cancer	*N* = 483	NR
	Li et al. (2021, 2022)^25^^,^^26^	Prospective observation	Germany	Multi-center	Ophthalmology: uveitis	*N* = 256	≥ 18
	Banerjee et al. (2022)^27^	Pilot test	USA	Academic medical center	Oncology: multiple myeloma	*N* = 15	50-81
System integration	Lizzio et al. (2019)^28^	System design	USA	Outpatient sports medicine	Orthopedics: general	NR	NR
	Hawley et al. (2021)^29^	System design	Canada	Mental health hospital	Mental health: major depressive disorder	NR	NR
	El Dahr et al. (2023)^30^	Pilot test	Canada	Community-based	Mental health: general	*N* = 66 pairs	9-13 (children)/NR (parents)
	Harry et al. (2024)^31^	Pilot test	USA	Pediatric rheumatology Clinic	Rheumatology: lupus	*N* = 17	11-19
	Kissler et al. (2024)^32^	Pilot test	USA	Outpatient and community	Obstetrics: latent phase of labor	*N* = 17/18	20-45
	Pack et al. (2025)^33^	Pilot test	USA	Primary care clinic	Geriatrics: polypharmacy	*N* = 64	≥ 60
Personalized insights	Espinoza et al. (2023)^34^	System design	USA	Pediatric academic Medical center	Endocrinology: pediatric Diabetes	*N* = 10+	≥ 7
	Turchioe et al. (2023)^10^	Impact evaluation	USA	Inpatient and outpatient	Cardiology: heart failure	*N* = 70	≥ 21
	MacMullen et al. (2024)^35^	System design	USA	Pediatric specialty program	Neurology: mitochondrial disease	*N* = 435/473	All ages
	Shi et al. (2025)^36^; Yu et al. (2025)^9^	System design	USA	Home-based care	Oncology: transitional care	*N* = 8+	NR

Abbreviations: NR, not reported. Sample size is presented as *N* (analyzed)/*N* (enrolled) to indicate participant flow and attrition where applicable. A plus sign (+) after a sample size number indicates that recruitment was ongoing at the time of publication.

**Figure 1.**
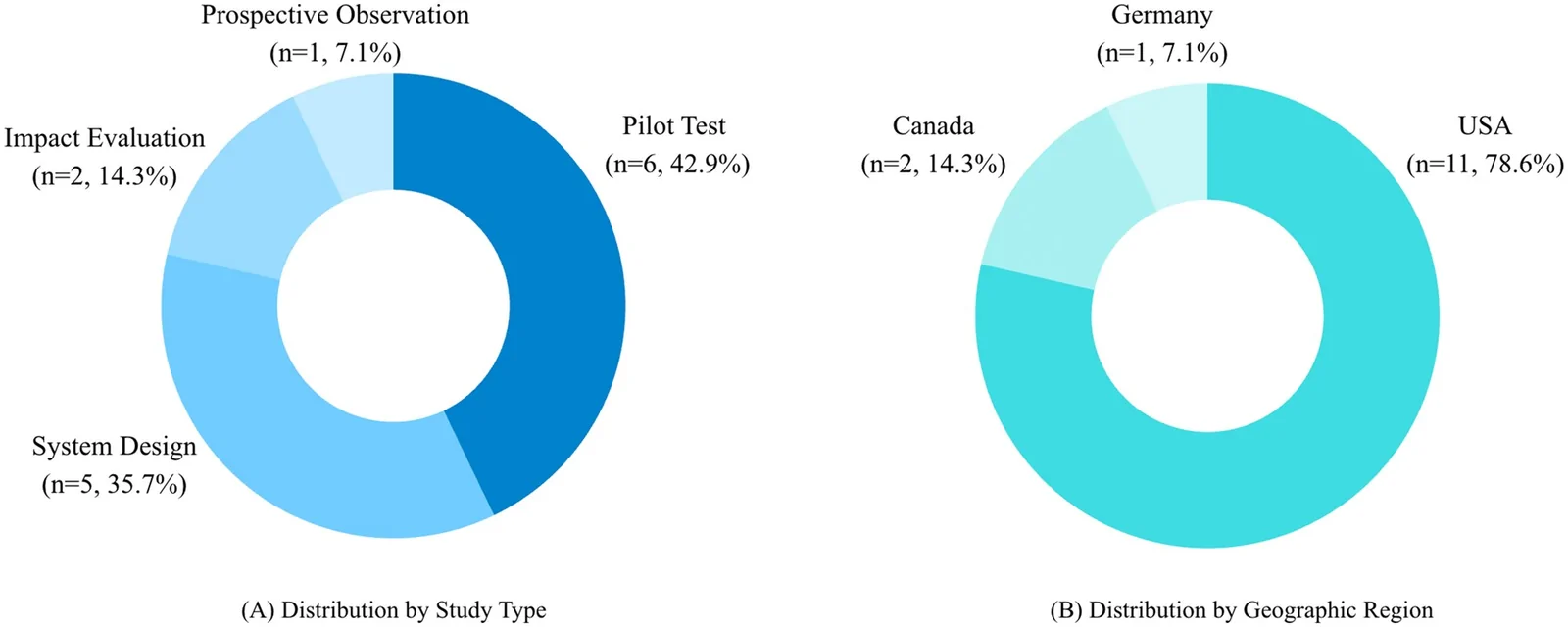
Distribution of included studies by study type and geographic region (*N* = 14). (A) The pie chart illustrates the breakdown of study designs, showing a predominance of early-stage research, specifically pilot tests and system design (*n* = 11). (B) The geographic distribution highlights that the majority of studies originated from the United States (*n* = 11).

**Figure 2.**
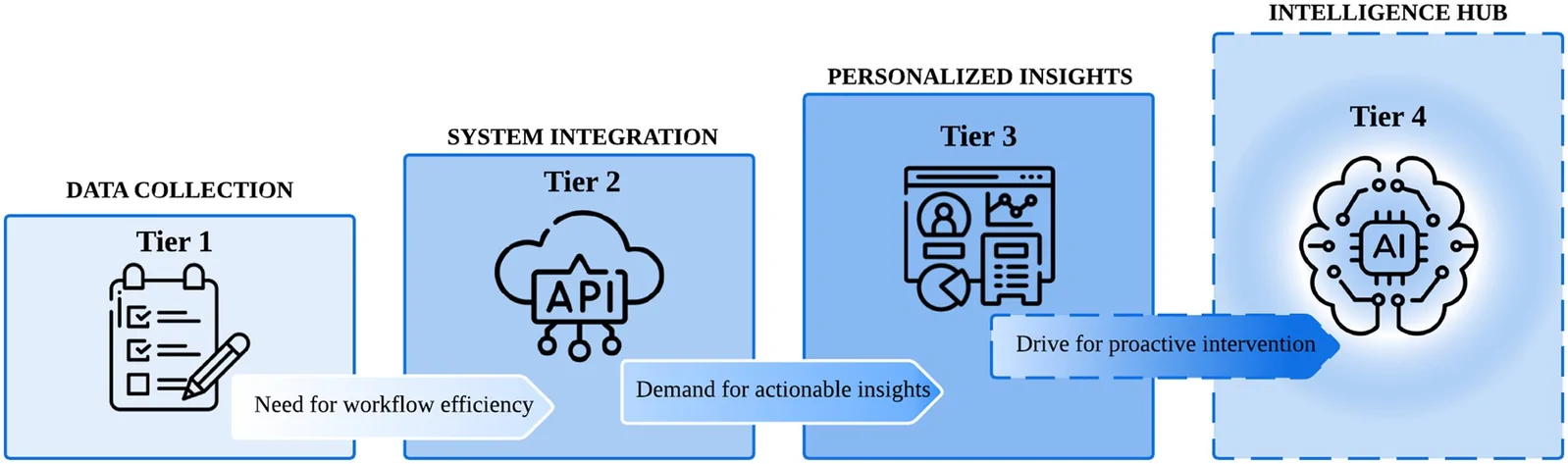
The 4-tier maturity model for REDCap-based patient-facing technology. The first 3 tiers (Tier 1-3) represent empirically derived stages from the integrative review, while the final tier, Tier 4, is conceptualized as a forward-looking extension and is therefore outlined using a dashed border.

To further dissect the landscape of REDCap-based PFTs, charts were created to visualize the methodological and geographic distribution of the included studies (Figure 1). These visualizations reveal 2 notable trends: (1) methodologically, most studies remain in the exploratory stage, with pilot tests and system design studies dominating the landscape (Figure 1A) and (2) the research effort is predominantly concentrated in the United States (Figure 1B).

### Description of the tailored maturity model

A thematic analysis of the included studies revealed that REDCap-based PFTs follow a clear evolutionary pathway, conceptualized as a maturity model comprising 3 fact-based tiers. This analysis synthesizes a comprehensive breakdown of the distinct characteristics and operational patterns observed across each tier of application (Table 2).

**Table 2.** Characteristics of the tiers in the tailored maturity model.

	Stated objective	Technical approach	Study outcomes	Implementation barriers
Data collection	Improve patient participation and accessibility Enhance data collection efficiency	Native REDCap web-surveys Automated email/SMS distribution Adaptive questionnaires	Enhanced data quality and accuracy Ensured data security and compliance Improved efficiency and cost-effectiveness	Siloed data workflows Deficient engagement Poor Scalability
System integration	Assess system feasibility and accessibility Optimize clinical and research workflows Enhance patient communication	External system integration: EHR/bi-directional SMS Advanced survey automation	High feasibility and acceptability Improved response metrics Enhanced platform adaptability	Lack of customization in automation Unsustainable engagement External service costs
Personalized insights	Empower patient and clinical decisions Integrate multi-dimensional health data Evaluate complex systems	Deep system integration Dynamic data visualization Novel AI augmentation	Unified data views Improved patient self-management Scalable system architecture	Specialized expertise requirement Data exchange vulnerabilities Practical usability challenges

Abbreviations: AI, artificial intelligence; EHR, electronic health records; PFT, patient-facing technology; SMS, short message service.

#### Tier 1: standalone data collection systems

Studies classified as Tier 1 used REDCap as a standalone data collection platform, primarily for capturing ePROs, with the core objectives of improving efficiency, increasing patient participation, and enhancing system accessibility.^23–27^ The technical approach typically involved distributing automated email or SMS links and employing adaptive questionnaires to minimize patient burden.^23^^,^^24^

Efficiency gains were evident, with the weekly research staff workload stabilizing at just 3 hours for ongoing operations following the initial 10-hour.^23^ Complementing these internal operational gains, patient participation also improved markedly, with 1 study reporting a rise in 30-day response rates from 80.7% to 94.0% after replacing paper-based methods.^24^ Furthermore, key outcomes included enhanced data quality, accuracy, and security, with implementations ensuring compliance with standards such as HIPAA.^23–26^ Despite the successes, commonly reported barriers at Tier 1 include siloed data from clinical workflows, which necessitates manual data handling,^23^^,^^25^^,^^26^ as well as inconsistent patient engagement and poor scalability of the standalone systems.^23^^,^^24^^,^^27^

#### Tier 2: system integration for workflow automation

Implementations in Tier 2 represent a significant evolution, with core objectives focused on assessing system feasibility, optimizing research workflows, and enhancing patient communication.^28–33^ These studies employed advanced survey automation and external system integration, primarily by connecting REDCap with a bidirectional SMS platform,^28^^,^^30^^,^^31^ such as Twilio, or by embedding assessments directly into clinical workflows, either via automated email and in-clinic tablet deployments^29^ or through EHR-tethered patient portals.^33^ Studies of Tier 2 consistently demonstrated high feasibility and acceptability among both patients and clinicians. For example, 1 study found that SMS notifications led to higher completion rates and faster response times than email, and that their integrated system demonstrated enhanced platform adaptability in managing complex logistical requirements, such as multiple time zones.^30^ Nonetheless, new implementation barriers emerged, including a lack of customization in automation that required extensive manual setup,^28^^,^^30^^,^^32^ challenges with unsustainable long-term patient engagement,^28^^,^^30–33^ and the introduction of external service costs.^30^^,^^31^

#### Tier 3: advanced visualization for actionable insights

The most mature stage to date, Tier 3, comprises technologically advanced applications designed to support patient and clinical decision-making by integrating multidimensional health data. The technical approach in these studies involved establishing data integration pipelines with health platforms, such as EHRs,^10^^,^^34^^,^^35^ using dynamic data visualization through interactive dashboards, and exploring novel AI augmentation features, including chatbots.^9^^,^^36^

The outcomes from Tier 3 included the creation of unified data views by extracting and combining datasets from both REDCap and EHRs within external analytics platforms,^34^^,^^35^ utilizing tools such as Tableau or Data Studio (formerly Looker Studio), and improved patient self-management facilitated by interactive dashboards.^9^^,^^34–36^ For patients, the primary benefit was the use of interactive dashboards that provided immediate, visual feedback on their reported health conditions over time, thereby empowering them in their own care.^10^ These implementations were also frequently described as having scalable system architectures, underscoring their potential for broader replication. This level of complexity, however, was associated with a set of barriers, including the requirement for advanced programming and systems integration capabilities,^9^^,^^10^^,^^34^^,^^36^ the emergence of data exchange vulnerabilities associated with third-party platforms,^9^^,^^36^ and practical usability challenges that necessitated user training.^9^^,^^10^^,^^36^

Notably, advanced implementations from our team’s work^9^^,^^36^ began to exhibit characteristics that extend beyond the typical scope of Tier 3. While still primarily focused on personalized insights, our PFT incorporated more advanced capabilities, including early-stage predictive analytics and AI architectures to enable automated feedback mechanisms. Specifically, the chatbot is designed to provide tailored summaries and suggestions based on patients’ tracked concerns and can personalize responses according to a patient’s medical history, current treatments, and reported concerns. These features hinted at a more profound functional discontinuity, representing the gap between passive systems and emerging automated support. To complete the model based on these findings and realize the full potential of patient-centered care, the fourth tier, the Intelligence Hub, is introduced, as shown in Figure 2.

## Discussion

A synthesis of the included studies indicates that PFTs are a promising approach to enhancing data quality and streamlining workflow processes. While the findings demonstrate potential benefits, they must be interpreted within the context of a nascent and exploratory research domain. This disparity between the anticipated impact of these strategies and the current maturity of the field provides a critical basis for the ensuing discussion.

### The current landscape: a young and rapidly evolving field

Our overall analysis situates these findings within a research area that is still developing and is limited to a specific geographical region. Most of the included studies were published since 2021 (*n* = 12). This recent surge, coupled with the predominance of pilot tests and system design studies (Figure 1A), confirms that the field is in a relatively early stage of development. The community’s primary focus has been on establishing technical viability and foundational approaches, rather than on demonstrating widespread clinical effectiveness or scalability. Furthermore, the heavy concentration of research in North America (Figure 1B) suggests that the current evidence base is shaped by the region’s specific healthcare systems, funding priorities, and the origins of the REDCap platform itself.

### The path forward: a maturity model as a roadmap

The tailored maturity model (Figure 2) serves not only as a classification framework but also as an actionable roadmap that elucidates the developmental trajectory of REDCap-based PFTs. Each tier represents a significant leap in technical capability and operational impact, illustrating the progression from simple data collectors to sophisticated, insight-driven platforms. This model consolidates our evidence-based findings (Tiers 1-3) and is further augmented through the conceptual extension provided by the Intelligence Hub (Tier 4). Ultimately, the model’s core utility is to help institutions and research teams benchmark their current position on this evolutionary path and inform the strategic planning required to advance toward greater maturity.

#### Tier 1: the foundational tier—digitizing data collection

Tier 1 represents the foundational level of digitalization in data capture. Its value lies in the transition from labor-intensive, error-prone paper processes to secure, compliant digital workflows. From a patient-centered perspective, this shift is critical, as it empowers patients to report health data in real time, thereby mitigating recall bias and improving data granularity.^9^^,^^32^^,^^36^ However, despite ensuring data quality, the utility of Tier 1 is inherently capped by its isolation. These systems typically function as disconnected data silos,^23^^,^^25^^,^^26^ creating a gap between effective data capture and clinical utility that necessitates the evolution to Tier 2.

#### Tier 2: the integration tier—automating workflows via system integration

The evolution to Tier 2 marks the pivotal transition from a standalone data collector to an integrated component of the broader clinical and research ecosystem. By integrating with external platforms (eg, EHRs or SMS services), the core objective shifts to overcoming data silos and automating workflows. This connectivity allows data to move automatically, reducing manual burden and enhancing timely communication.^29^^,^^30^ Yet, the barriers at this tier reveal a critical limitation: reliance on rigid, rule-based automation. The necessity for extensive manual setup and predefined instructions^28^^,^^30–33^ indicates that merely connecting systems to automate processes is not the goal; the data lacks the capacity to be transformed into clinical knowledge or patient-facing insights, underscoring the need for the next level of maturity: Tier 3.

#### Tier 3: the insight tier—generating knowledge from personalized insights

Tier 3 represents a fundamental paradigm shift from operational automation to the generation of actionable knowledge. By synthesizing previously siloed data streams into unified visualizations, this tier strategically empowers patients with contextualized feedback, transforming them from passive data providers into informed partners in their own care. While highly valuable, Tier 3 systems face barriers similar to those in enterprise software, including resource-intensive development and usability challenges. Critically, despite their sophistication, the system logic remains fundamentally reactive. The reliance on human interpretation to translate visualizations into interventions creates a functional bottleneck, motivating the final evolutionary leap to Tier 4.

#### Tier 4: the intelligent tier—transforming reactiveness into proactive care

To overcome this functional bottleneck, Tier 4 represents the ultimate evolutionary leap, moving the REDCap-based PFTs from a knowledge generator to an autonomous, proactive intervention partner. Tier 4 completes the model’s goal of achieving the full potential of patient-centered care.

The conceptualization of this tier is primarily supported by the PFT developed by our team,^9^^,^^36^ which served as a practical blueprint for the transition. This PFT demonstrated the technical feasibility of integrating early-stage predictive analytics and advanced AI architectures within the REDCap ecosystem. Unlike simple dashboards that merely visualize data, the Intelligence Hub leverages AI to implement an autonomous control loop where the system can analyze the unified data view, identify risk factors in real time, and automatically trigger tailored guidance or alerts.

Functionally, the Intelligence Hub is defined by its ability to provide decision support. The system’s core value lies in transforming patient-reported data and clinical records into actionable recommendations, which are delivered directly to patients via personalized chat. Although this evolution turns the PFT from a passive data entry tool into an intelligent collaborator, enabling proactive risk management and timely care, it may face stricter regulatory and quality requirements, such as FDA review, system validation, and change management. REDCap-based PFTs predominantly operate in research or operational support contexts rather than as broadly scalable, direct clinical interventions. Tier 4 is paramount for the long-term sustainability and scalability of PFTs, as automated, intelligent support can significantly reduce the resource-intensive human oversight required by Tier 3 implementations.

### Navigating the roadmap: implications and future directions

In this study, we define REDCap-based PFTs as tools in which REDCap underpins patient interaction, data capture, or communication. These span 2 categories: (1) configurations that rely primarily on built-in REDCap capabilities and its mobile solutions—such as surveys, SMS, and Alerts & Notifications—which typically correspond to lower-complexity implementations in Tiers 1-2 and, when combined with more advanced longitudinal workflows, Tier 3 and (2) external applications that interface with REDCap via the API, using REDCap primarily as a secure backend database while providing enhanced patient- or administrator-facing interfaces. These API solutions may range from Tiers 1-3, and sometimes Tier 4, depending on the number of external systems integrated, including but not limited to EHRs, patient portals, registries, messaging, and analytics platforms, as well as the logic complexity of these systems.

The tailored maturity model (Figure 2) presented in this review offers several key implications for both practice and future research. It is aimed to enable research and clinical teams to benchmark the current state of their REDCap PFTs and identify the specific challenges and opportunities associated with their tier. The model’s clear delineation of capabilities and barriers at each stage can inform institutional strategic planning, offering a structured framework for evaluating the investment benefits of advancing system maturity. Beyond its utility for targeted user groups and developers, the model also illuminates a path for REDCap’s own evolution. Specifically, the heavy dependence on external visualization tools and the substantial development costs recognized as challenges in Tiers 2 and 3 indicate that the REDCap development team should strengthen native data analysis and visualization functionalities to accommodate the growing requirements for integrated, real-time data management. Moving forward, this review advocates for a research agenda centered on 3 pivotal, interconnected domains.

1. *Exploring the Tier 4 frontier of AI-driven communication:* The Intelligence Hub represents the highest value for patient care. Future studies should build upon the architectural concepts to rigorously validate how proactive AI-driven chatbots and autonomous control loops impact measurable patient health outcomes, ensuring the transition from reactive data consumption to intelligent, automated care partnerships.
2. *Lowering technical barriers through standardization:* Custom integrations in Tiers 2 and 3 are resource-intensive, hindering widespread use. Future work should focus on creating standardized, reusable modules to connect REDCap with other systems. These modules could include External Modules Framework components, templates, and configuration bundles that support common PFT patterns and interoperability pipelines linking REDCap to external systems. Such pipeline enhancements, for example, through Clinical Data Interoperability Services (CDIS), could facilitate access to data in EHRs and patient portals via Fast Healthcare Interoperability Resources (FHIR) APIs, enable automated data flows to analytics and visualization environments such as Data Studio, Tableau, or Power BI, and streamline integration with registries and messaging platforms for real-time alerts. This approach is expected to lower adoption barriers, enable flexible configurations, and provide a unified data view for Tier 4 systems. Ultimately, by coupling these external interoperability pipelines with REDCap’s native capabilities, the future of PFTs encompasses not merely digital data collection but also adaptive, automated, and feedback-oriented systems. For instance, systems that utilize branching logic to customize inquiries, generate alerts and notifications for follow-up, employ APIs to display trends in dashboards, and utilize file repositories or action tags to connect patients or clinicians with the subsequent suitable resource or action.^36^
3. *Validating clinical impact with rigorous methods:* While Tier 3 studies report improved patient self-management as a key outcome, evidence for tangible clinical impact remains uncertain. More rigorous research, including randomized controlled trials, is needed to better understand the impact of these advanced, patient-facing dashboards on measurable health outcomes and to confirm the clinical utility of the insights generated by Tier 3, as well as the communication triggered by Tier 4.

### Scoping the roadmap: strengths and limitations of this review

This review adheres to established standard guidelines. By anchoring the maturity model in a systematic search and synthesis of peer-reviewed literature, the review presents its findings within an evidence-based framework that delineates the current landscape of REDCap-based PFTs. This methodology ensures that each model tier is supported by real-world examples and reported outcomes.

However, several limitations should be considered when interpreting these findings. First, a key limitation of this review is the possible underreporting of REDCap-based PFTs in the literature. While these tools are widely used for ePRO collection and participant interaction, their technical details are rarely described. Most articles focus on study outcomes rather than on PFT design, development, scalability, or sustainability. Consequently, our sample may represent a small, biased subset of real-world deployments, limiting the generalizability of our findings. Future work should promote more detailed reporting, develop specific guidelines, and include surveys or registries of operational projects not formally published. Second, the analysis is constrained by significant heterogeneity in study designs and populations, which is common in this emerging field. We did not perform a formal quality assessment, as the review concentrated on implementation evidence and a conceptual model rather than clinical effectiveness. Furthermore, the absence of 2 fully independent reviewers at all stages may have introduced reviewer or classification bias. Third, while AI-augmented PFTs (eg, chatbots, automated summaries) show promise, their application in REDCap-based systems is constrained primarily by legal, ethical, and governance issues rather than technical feasibility. Key challenges include data protection, especially when integrating external large language models, algorithmic transparency, and clinical liability. Safe deployment ultimately demands clear policies, rigorous validation, and regulatory review.

## Conclusion

In summary, REDCap-based PFTs demonstrate strong potential to enhance data quality and workflow efficiency, but their benefits remain constrained by the field’s early stage of development. As outlined in our tailored maturity model, bridging this gap will require ongoing and more comprehensive research and implementation efforts to turn these promising strategies into sustainable, evidence-based practices.

## Supplementary Material

ooag176_Supplementary_Data

## Data Availability

No new data were generated or analyzed in support of this research.
