# Supplementary material for: A tailored maturity model for REDCap-based patient-facing technology: revealing development stages and guiding future evolution
Source: JAMIA Open. 2026 Sep 10;9(5):ooag176. doi: 10.1093/jamiaopen/ooag176 (PMC13561520; doi:10.1093/jamiaopen/ooag176)
Supplement: Supplementary file 1 [file ooag176_supplementary_data.docx]

**APPENDICES**

**Appendix A: Detailed Search Strategy**

The search was first performed in December 2024 and updated in June 2026. The search was limited to articles published in English and was not restricted by publication date. The following table details the search terms used for each database. Within each database, terms from Concept A and Concept B were combined using the “AND” operator, while terms within each concept were combined using the “OR” operator.

**Table A1. Search Strategy by Database and Concept**

| Database | Concept A: Platform | Concept B: Application |
| --- | --- | --- |
| PubMed | (“REDCap” OR “Research Electronic Data Capture” OR “MyCap”) | (“patient reported outcome*” OR “patient facing technology” OR “ePRO” OR “patient portal” OR “Patient-Generated Health Data”) |
| Web of Science | TS=(“REDCap” OR “Research Electronic Data Capture” OR “MyCap”) | TS=(“patient reported outcome*” OR “patient facing technology” OR “ePRO” OR “patient portal” OR “Patient-Generated Health Data”) |
| Embase | (‘REDCap’ OR ‘Research Electronic Data Capture’ OR ‘MyCap’):ti,ab,kw | (‘patient reported outcome*’ OR ‘patient facing technology’ OR ‘ePRO’ OR ‘patient portal’ OR ‘Patient-Generated Health Data’):ti,ab,kw |

**Appendix B:** **Literature Management and Data Extraction Tools**

The following table details the software and plugins utilized to ensure a systematic and transparent process for literature management and data synthesis in this review.

**Table B1. Tools Used for Literature Management and Analysis**

| Tool | Purpose in This Review |
| --- | --- |
| Zotero (v9.0.4) | Reference Management & Citation |
| Zotplicate (Zotero Plugin) | Advanced De-duplication |
| Notion | Data Extraction & Synthesis |
| Notero (Zotero Plugin) | Zotero-Notion Synchronization |

**Appendix C:** **PRISMA 2020 Flow Diagram of the Study Selection Process**

**Figure C1: The PRISMA 2020 Flow Diagram of the Study Selection Process.**
